# Human-AI teams—Challenges for a team-centered AI at work

**DOI:** 10.3389/frai.2023.1252897

**Published:** 2023-09-27

**Authors:** Vera Hagemann, Michèle Rieth, Amrita Suresh, Frank Kirchner

**Affiliations:** ^1^Business Psychology and Human Resources, Faculty of Business Studies and Economics, University of Bremen, Bremen, Germany; ^2^Robotics Research Group, Faculty of Mathematics and Computer Science, University of Bremen, Bremen, Germany; ^3^DFKI GmbH, Robotics Innovation Center, Bremen, Germany

**Keywords:** human-agent teaming, hybrid multi-team systems, cooperation, communication, teamwork, integrative artificial intelligence

## Abstract

As part of the Special Issue topic “Human-Centered AI at Work: Common Ground in Theories and Methods,” we present a perspective article that looks at human-AI teamwork from a team-centered AI perspective, i. e., we highlight important design aspects that the technology needs to fulfill in order to be accepted by humans and to be fully utilized in the role of a team member in teamwork. Drawing from the model of an idealized teamwork process, we discuss the teamwork requirements for successful human-AI teaming in interdependent and complex work domains, including e.g., responsiveness, situation awareness, and flexible decision-making. We emphasize the need for team-centered AI that aligns goals, communication, and decision making with humans, and outline the requirements for such team-centered AI from a technical perspective, such as cognitive competence, reinforcement learning, and semantic communication. In doing so, we highlight the challenges and open questions associated with its implementation that need to be solved in order to enable effective human-AI teaming.

## 1. Introduction

In the future, Mars is a target for long-duration space missions (Salas et al., 2015). Both governments and private space industries are fascinated by the Red Planet, and are aiming to send teams of astronauts on a mission to Mars in the late 2030's (Buchanan, 2017; NASA, 2017). For successful survival and operation on Mars, a habitat with intelligent systems, such as integrative Artificial Intelligence (Kirchner, 2020), and robots (e.g., for outdoor operations), are indispensable, among other things. To avoid unnecessary exposure to radiation the crew will be in the habitat most of the time. There, they will collaborate with technical systems with capabilities that are more like the cognitive abilities of humans compared to previous support systems. Advancements in Machine Learning and Artificial Intelligence (AI) have led to the development of systems that can handle uncertainties, adjust to changing situations, and make intelligent decisions independently (O'Neill et al., 2022). Intelligent autonomous agents can either exist as virtual entities or can embody a physical system such as a robot. Although much of the decision-making paradigm may be similar in both cases, the physical spatio-temporal constraints of robots must be properly considered in their decisions (Kabir et al., 2019). In the given context, autonomous agents perform tasks such as adaptively controlling light, temperature, and oxygen levels. In addition, they can gather important information about the outdoor environment and guide the crew's task planning by telling them when, for example, an outdoor mission is most advantageous due to weather conditions such as isotope storms. Additionally, for outdoor activities, multi-robot teams (Cordes et al., 2010) will facilitate efficient exploration in areas of low accessibility, transportation of materials, and analysis and transmission of information to the human crew.

In the previously described scenario, we are concerned with human-AI teaming (cf. Schecter et al., 2022), also often referred to as human-agent teaming (cf. Schneider et al., 2021) or human autonomy teaming (cf. O'Neill et al., 2022) (all abbreviated as HAT). Those are systems, in which humans and intelligent, autonomous agents work interdependently toward common goals (O'Neill et al., 2022). These forms of hybrid teamwork (cf. Schwartz et al., 2016a,b) are already present in some industries and workplaces and are becoming more and more relevant, for example in aviation, civil protection, firefighting or medicine. They provide opportunities for increased safety at work and productivity, thus supporting human and organizational performance.

A well-known example from the International Space Station is the astronaut assistant CIMON-2 (Crew Interactive MObile companioN), which has already worked with the astronauts. CIMON-2 is controlled by voice and aims to support astronauts primarily in their workload of experiments, maintenance, and repair work. Astronauts can also activate linguistic emotion analysis, so that the agent can respond empathically to its conversation partners (DLR, 2020). Another example of AI at work is the chatbot CARL (Cognitive Advisor for Interactive User Relationship and Continuous Learning), which has been in use in the human resources department of Siemens AG. CARL can provide information on a wide range of human resources topics and thus serves as a direct point of contact for all employees. Also, the human resources Shared Service Experts themselves use CARL as a source of information in their work. CARL understands, advises, and guides and is used extensively within the company. Carl has been positively received by the employees as well as the human resources experts and leads to a facilitation in the work like a colleague (IBM and ver.di, 2020). Artificial agents are also used in the medical sector, for example, when nurses and robots collaborate efficiently in the Emergency Department during high workload situations, such as resuscitation or surgery.

The question that emerges is how to effectively design such a novel form of teamwork that fully meets the needs of humans in a successful teamwork process (Seeber et al., 2020; Rieth and Hagemann, 2022). This perspective thus highlights the role of AI interacting with humans in a team instead of only using a normal high developed technology. Consequently, this perspective aims toward a comprehensive and interdisciplinary exploration of the key factors that contribute to successful collaboration between humans and AI. We (1) illustrate the requirements for successful teamwork in interdependent and complex work domains based on the model of an idealized teamwork process, (2) identify the implications of these requirements for successful human-AI teaming, and (3) outline the requirements for AI to be team-centered from a technical perspective. Our goal is to draw attention to the teamwork-related requirements to enable effective human-AI teaming, also in hybrid multi-team systems, and at the same time to create awareness of what this means for the design of technology.

## 2. Human-AI multi-team systems

As described above, the question arises as to what aspects need to be considered in human-centered AI in teamwork, both in terms of the human crew and the “team” of artificial agents to achieve effective and safe team performance. Imagine a scenario for major disasters on earth. Here we will not only have a team with one or two humans and one agent, but several teams of people, e.g., police, fire fighters, rescue services, and several agents, e.g., assistance system in the control center, robots in buildings and drones in the air, who must communicate and collaborate successfully.

Thus, HATs also exist in a larger context and work in dependence with other teams. These human-AI multi teams are called hybrid multi-team systems (HMTS) and refer to “multiple teams consisting of n-number of humans and n-number of semi-autonomous agents [i.e., AI] having interdependence relationships with each other” (Schraagen et al., 2022, p. 202). They consist of sub-teams, with each individual and team striving to achieve hierarchically structured goals. Lower-level goals require coordination processes within a single team and higher-level goals require coordination with other teams. Their interaction is shaped by the varying degrees of task interdependencies between the sub-teams (Zaccaro et al., 2012). HMTS highlight the complexity of the overall teamwork situation, as sub-teams consist of humans, of agents and of humans and agents. Therefore, teamwork relevant constructs such as communication (Salas et al., 2005), building and maintaining an effective situation awareness (Endsley, 1999) and shared mental models (Mathieu et al., 2000) as well as decision making (Waller et al., 2004) will not only be of high relevance in the human crew, but also in the AI teams (Schwartz et al., 2016b) as well as in the human-AI teams (cf. e.g., Carter-Browne et al., 2021; Stowers et al., 2021; National Academies of Sciences, Engineering, and Medicine, 2022; O'Neill et al., 2022; Rieth and Hagemann, 2022).

**Figure 1:**
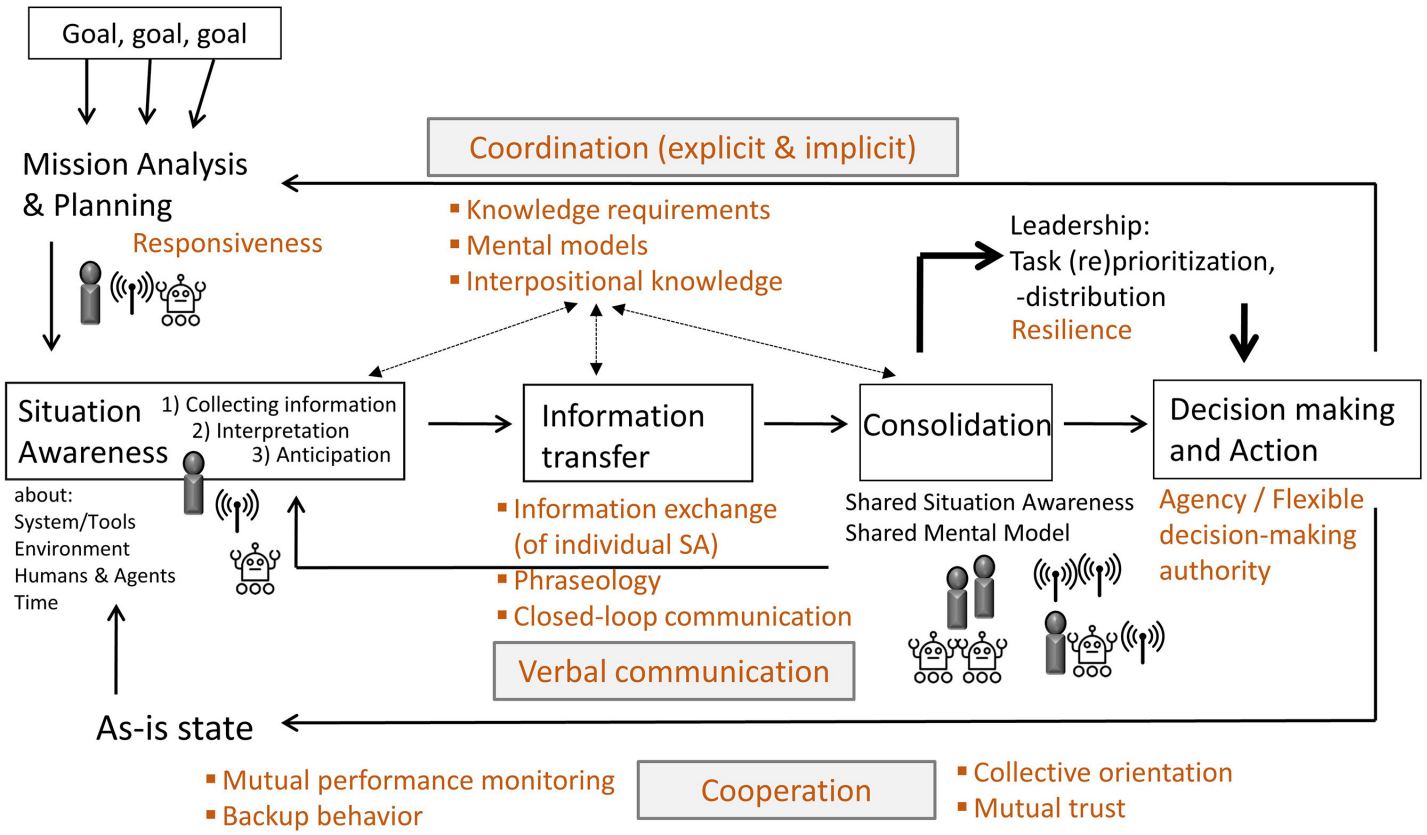
Model of an idealized teamwork process of hybrid multi-team systems. Adapted from Hagemann and Kluge (2017), licensed under CC BY 4.0.

To date, research has focused on individual facets of successful HAT, ignoring the Input-Process-Output (IPO) framework (Hackman, 1987) in teamwork research (cf. O'Neill et al., 2023) which acknowledges the pivotal role of group processes (e.g., shared mental models or communication) in converting inputs (e.g., autonomy or task) into desired outcomes (e.g., team performance or work satisfaction). Often, the focus is on single aspects such as trust (Lyons et al., 2022), agent autonomy (Ulfert et al., 2022), shared mental models (Andrews et al., 2023), or speech (Bogg et al., 2021). Examining individual facets is important to understand human-AI teaming, yet we would like to point out that *successful teamwork* does not consist of individual components *per se*, but rather the big picture, i.e., *the interaction of inputs, processes and outcomes*. Thus, we would like to think of a *teamwork-centered AI holistically* and *discuss relevant aspects for a successful human-AI teaming from a psychological and technical perspective using the model of the idealized teamwork process* (Hagemann and Kluge, 2017; see the black elements in Figure 1).

### 2.1. Teamwork requirements in human-AI teaming

The cognitive requirements for effective teamwork and the team process demands are consolidated within the model of an idealized teamwork process (Kluge et al., 2014; Hagemann and Kluge, 2017). Figure 1 shows an adapted version of this model. The black elements in the model are from the original model by Hagemann and Kluge (2017). The brown elements of the model are additions which, based on literature analyses, are essential for team-centered AI addressing human needs and thus for successful human-AI teaming. These elements will now be discussed in more detail in the course of this article. Following the IPO model, our proposed model does not focus on solely a certain component of the model, such as only the input, but holistically all three components. Central elements of the model are situation awareness, information transfer, consolidation of individual mental models, leadership, and decision making (for a detailed explanation see Hagemann and Kluge, 2017). Human-AI teams are responsible for reaching specific goals (see top left of model), for example, search for, transport, and care for injured persons during a large-scale emergency, as well as extinguishing fires. Based on the overall goals, various sub goals exist for the all-human teams, the agent teams, and the human-AI teams that will be identified at the beginning of the teamwork process and communicated within the HMTS. For routine situations, there will be standard operating procedures known by all humans and agents. However, it becomes challenging for novel or unforeseen situations for which standard operating procedures do not yet exist. Here, an effective start requires an intensive exchange of mission analysis, goal specification, and strategy formulation, which are important teamwork processes occurring during planning activities (cf. Marks et al., 2001). Such planning activities are a major challenge, especially in multi-team systems, since between-team coordination is more difficult to achieve than within-team coordination, but it is also more important for effective multi-team system teamwork (Schraagen et al., 2022). Thus, the *responsiveness* of the agents will be important for a team-centered AI (see upper left corner in the model), meaning that the *agents are able to align their goals and interaction strategies to the shifting goals and intentions of others as well as the environment* (Lyons et al., 2022).

As depicted in our model, the defined goals provide the starting position for all teams building an effective situation awareness, which is important for successful collaboration within teams (Endsley, 1999; Flin et al., 2008). Situation awareness means collecting information from systems, tools, humans, agents, and environments, interpreting this information and anticipating future states. The continuous assessment of situations by all humans and agents is important, as they work independently as well as interdependently and each team needs to achieve a correct situation awareness and to share it within the HMTS. High-performing teams have been shown to spend more time sharing information and less time deciding on a plan, for example (Uitdewilligen and Waller, 2018). This implies the importance of a *sound and comprehensive situation awareness between humans and agents* (cf. McNeese et al., 2021b) and an accompanying goal-oriented and continuous exchange of information in HMTS. For developing a shared situation awareness, the information transfer focuses on sending and receiving single situation awareness between team members. *Aligned phraseology* between humans and agents (i.e. using shared language and terminology) and *closed-loop communication* (i.e. verifying accurate message understanding through feedback: statement, repetition, reconfirmation; Salas et al., 2005) are essential for effective teamwork. However, possible effects of closed-loop communication have not yet been investigated in a HAT. Thus, it is not clear, for example, whether this form of communication is more likely to be considered disruptive in joint work and whether it should be used only in specific situations, such as when performing particularly important or sensitive tasks. Nevertheless, these requirements for communication in a HAT are important to consider for successful teamwork, as it has been shown that performance and perception of teamwork are significantly higher with verbal communication in a HAT (Bogg et al., 2021). Therefore, for a team-centered AI, *agents should communicate quite naturally with human team members in verbal language*.

Expectations of all humans and agents based on their mental models and interpositional knowledge impact the situation awareness, the information transfer, and the consolidation phases. Mental models are cognitive representations of system states, tasks, and processes, for example, and help humans and agents to describe, explain, and predict situations (Mathieu et al., 2000). Interpositional knowledge refers to an understanding of the tasks and needs of all team members to develop an understanding of the impact of one's actions on the actions of other team members and vice versa. It lays a foundation for understanding the information needs of others and the assistance they require (Smith-Jentsch et al., 2001). Interpositional knowledge and mental models are important prerequisites for effective coordination in HMTS, i.e., temporally and spatially appropriately orchestrated actions (Andrews et al., 2023). Thus, a *fully comprehensive and up-to-date mental model of the agents about the tasks and needs of the other human and artificial team members is highly relevant for team-centered AI*.

Based on effective information transfer, a common understanding of tasks, tools, procedures, and competencies of all team members is developed in the consolidation phase in terms of shared mental models. These shared knowledge structures help teams adapt quickly to changes during high workload situations (Waller et al., 2004) and increase their performance (Mathieu et al., 2000). The advantage of shared mental models is that HMTS can shift from time-consuming explicit coordination to implicit coordination in such situations (cf. Schneider et al., 2021). For example, observable behaviors or explicit statements may cause the agent to exhibit appropriate behavior, such as a robot observes that the human has reached a certain point in the experiment and is already preparing the materials that the human will need in the next step. Accordingly, a team-centered AI must be able to *coordinate with the humans in the team not only explicitly, but also implicitly*. In addition, *the agents in a HMTS must be able to detect when there is a breakdown in collaboration between humans and agents, or between the different agents*, and intervene so that they can explicitly coordinate again.

As a result of the consolidation phase, the HMTS or leading humans and agents need to make decisions to take actions. Thus, it is important that the artificial agents have *agency*, i.e., they can have control over their actions and the decision authority to execute these actions (Lyons et al., 2022). For an effective collaboration of humans and agents, the HMTS needs a *flexible decision-making authority*, that is, authority dynamically shifting among the humans and agents in response to complex and changing situations (Calhoun, 2022; Schraagen et al., 2022). Requirements in this phase include task prioritization and distribution as well as re-prioritization and distribution of tasks according to changes in the situation or plan (Waller et al., 2004). The *resilience* of the system is thus also important for team-centered AI, so that the agents can adapt to changing processes and tasks (Lyons et al., 2022). In this phase, it is very important that the *agents can interpret the statements of all the others and continue to think about the situation together with the humans*. Only in this way can HMTS be as successful as only human high-performing teams. That is due to the fact that in the decision-making phase high-performing teams compared to low performing teams use more interpretation-interpretation sharing sequences: the process involves an initial statement made by one human or agent, followed by an interpretative response from another agent, leading to a subsequent statement by the first agent that builds upon and expands the reasoning and thus build a collective sensemaking (Uitdewilligen and Waller, 2018).

The result of decision-making and action flows back into individual situation awareness and the original goals are compared with the as-is state achieved. This model of a continuously idealized teamwork process includes diverse feedback loops that enable a HMTS to adapt to changing environments and goals. For the described processes to be successfully completed, cooperation is required within the HMTS. This includes, for example, *mutual performance monitoring*, in which humans and agents keep track of each other while performing their own tasks to detect and prevent possible mistakes at an early stage (Paoletti et al., 2021). Cooperation also requires *backup behavior* in the team, i.e., the discretionary help from other human or artificial team members as well as a distinct *collective orientation* of all members (Salas et al., 2005; Hagemann et al., 2021; Paoletti et al., 2021). For team-centered AI, the agents must be able to provide this support behavior for the other team members. A successful pass through the teamwork process model also depends on the *trust* of each team member (Hagemann and Kluge, 2017; McNeese et al., 2021a). Important for the trust of humans in agents is a reliable performance, i.e., as few to no errors as possible (Hoff and Bashir, 2015; Lyons et al., 2022). Nevertheless, the agent should not only be particularly reliable, but for a team-centered AI it should also be able *to turn to all members of the HMTS in new situations and request an exchange because it cannot get on by itself*.

### 2.2. Technical requirement for artificial team members

Increased autonomy enables agents to *make decisions independently* in different situations, i.e., to develop *situational awareness*, even in situations where there is only a limited possibility of human intervention. For agents to be part of HMTS, it is mandatory to achieve a level of *cognitive competence* that allows them to grasp the intentions of their teammates (Demiris, 2007; Trick et al., 2019). This claim is much easier said than done as it requires the existence of mental models in agents that are comparable to the models that humans rely on, especially if they exchange information. However, such models cannot just be preprogrammed and then implanted into agents. One reason for this is that the process by which mental models are created in humans is still a subject under investigation (Westbrook, 2006; Tabrez et al., 2020). Even though this cognitive competence is required for team-centricity, this process is difficult to reproduce artificially. On the other hand, there is usually not just a single isolated model (or brain process) that generates human behavior, but rather an ensemble of models that are active at any given time and influence the observable outcome. Compared to humans that function based on cognitive decision-making, intuition, etc., machines are digitized, and act based on experience, their understanding of the current situation, and prediction models. These models must improve over time, based on a limited set of prerecorded data to move toward more accurate, robust systems. Thus, for future developments in HATs it would be important to design models with higher *predictive power*, which we define by how well the model can predict the outcome of its decisions based on the situation, experience and team behavior (see also Raileanu et al., 2018).

Moreover, agents must be competent and empowered to make decisions when needed, without having to wait for instructions from humans, especially in extreme environments where humans must adapt to particular conditions (Hambuchen et al., 2021), and resources are scarce. System *resilience* is also of high importance as the consequences of failure, on either side (human or agent) could be catastrophic. Whenever there is a potential threat to human lives, HMTS can prove more effective compared to homogeneous human or AI teams. During search and rescue operations on Earth (Govindarajan et al., 2016), *responsiveness, coordination, and effective communication* are crucial requirements for HMTS. Therefore, through teleoperation and on-site collaboration, HATs are able to mitigate the impact after a disaster. HATs can also be witnessed in modern medical applications that demand cooperation and high degrees of precision. For example, nurses and robots in the Emergency Department can efficiently handle high workloads, and safety-critical procedures like surgery and resuscitation, using a new *reinforcement learning* system design (Taylor, 2021). Reinforcement learning is a class of machine learning algorithms, wherein the agent receives either a reward or a penalty depending on the favorability of the outcome of a particular action.

In HATs of the future, we will thus have to work with agents that can learn over time to adjust to human behavior and shape the models of the environment and of other team members over time. This learning approach will enable the agents to exchange substantial information even with very few bits or in other words content and meaning will be exchangeable between humans and agents rather than bits and bytes. This process, also known as *semantic communication*, is currently under investigation by different teams from a more information theoretic approach over *symbolic reasoning* to an approach that is called *integrative artificial intelligence* (Kirchner, 2020). Beck et al. (2023) approach this problem by modeling semantic information as hidden random variables to achieve reliable communication under limited resources. This is a valuable step toward adapting to the problem of communication losses and latencies in applications like space, and exploration in remote areas. In a HAT setup, it is important to make some decisions regarding the nature of the team, either a priori or dynamically. Like pure human systems, assigning specific roles and defining hierarchies among agents in a team and between teams can enhance the overall mission strategy. Role-based task allocation is especially useful when the team consists of heterogeneous (Dettmann et al., 2022) and (or) reconfigurable (Roehr et al., 2014) agents. In HMTS, having every member trying to communicate with every other member is highly impractical, resource intensive and chaotic. This issue is further complicated when all members are authorized to act as they will. Implementing an *organized hierarchical team structure* (Vezhnevets et al., 2017) is therefore imperative for a team-centered AI successfully collaborating with humans.

To achieve seamless interaction between humans and agents, the latter must display behavioral traits that are acceptable to humans. An agent is truly team-centered when it can intelligently adapt to the situation and team requirements, in a *team-oriented* (Salas et al., 2005), rather than a dominant or submissive manner. Agents need to achieve predictive capabilities for other teammates and the environment to account for variation, as in Raileanu et al. (2018). In the autonomous vehicle domain, it is crucial that the vehicle can accurately predict the behavior of pedestrians and others to enable seamless navigation (Rhinehart et al., 2019). According to Teahan (2010), behavior is defined by how an agent acts while interacting with its environment. Interaction entails *communication* which can be either verbal or non-verbal. An interesting aspect will be to investigate deeper into large language models, like ChatGPT and to find out if these approaches can be extended to general interactive behavior (Park et al., 2023) instead of just text and images. Apart from language, verbal communication is also characterized by the acoustics of the voice, and style of speech. Moreover, movement is a fundamental component that defines the behavior of any team member. Depending on design, agents are already capable of performing and recognizing gestures (Wang et al., 2019; Xia et al., 2019) and emotions (Arriaga et al., 2017). Motion analyses have shown that the intention behind performing an action is intrinsically embedded in the style of movement, for instance, in the dynamics of the arm (Niewiadomski et al., 2021; Gutzeit and Kirchner, 2022). In all the scenarios, one of the biggest challenges faced by HMTS is the *trustworthiness* of the team. Cooperation requires building trust-based relationships between the team members. Bazela and Graczak (2023) evaluated, among other factors, “*the team's willingness to consider it* [the Kalman autonomous rover—an astronaut assistant] *a partially conscious team member*” (p. 369). The opposite also holds true. Agents must maintain a *high trust factor* of their human teammates, i.e., be able to trust humans, as this factor has a significant influence on the decision-making process (Chen et al., 2018). For instance, the agent's trust factor can be improved by means of reliable communication when the human switches strategies. From a technical perspective, humans are the chaos factor in the HAT equation and even though this can be modeled to a certain extent on the agent side, an effective collaboration largely depends on the predictability of human actions. A summary of all the requirements mentioned for team-centered successful human-AI teaming addressing human needs can be found with definitions of these and example references in the Supplementary Table 1.

## 3. Conclusion

The aim of this contribution is to discuss the central teamwork facets for successful HATs in an interdisciplinary way. Starting from a psychological perspective addressing the human needs, the importance of team-centered AI is revealed. However, its technical feasibility is challenging. It is an open problem from a standpoint of technical cognition if AI systems can ever be regarded and/or accepted as actual team members as this poses a very fundamental question of AI. This question refers to the challenge to replicate intelligence in technical systems as we only know it from human systems. This is an old and long-standing question that has been addressed by Turing (1948) in his famous paper on “*intelligent machinery*” already in the early last century. As a mathematician he concluded that it is not possible to build such systems *ad hoc*. One loophole that he identified in this paper is to create highly articulated robotic systems that learn—in an *open-ended process*- from the inter*action* with a *real*-world environment. It is his assumption that somewhere along this process, which is *open-ended*, some of the features that we associate with intelligence may emerge and thus the resulting system will eventually be able to simulate intelligence well enough such that it will be regarded as intelligent by humans. If this is actually feasible has never been tested but could be a worthwhile experiment to perform with technologies of the 21st century. However, for the meantime, teamwork attributes like *responsiveness, situation awareness, closed-loop communication, mental models, and decision making* remain to be buzz words in this context and are technical features that we will be able to implement to a limited extent into technical systems in order to enable these systems to act as valuable tools for humans in well-defined environments and contexts. But whether this will qualify the agents as team members is unknown so far (cf. also Rieth and Hagemann, 2022). This would in fact require a much deeper understanding of the processes that enable cognition in human systems as we have it today and even if we had that understanding, it will still be an open question if the understanding of mental processes is also a blueprint or design approach to achieve the same in technical systems. Overall, the manuscript provides insights into the team-centered requirements for effective collaboration in HATs and underscores the importance of considering teamwork-related factors in the design of technology. Our proposed guidelines can be used to design and evaluate future concrete interactive systems. In the experimental testing of the single facets discussed for a truly team-centered and successful HAT, which considers the needs of the humans in the HAT, many highly specific further research questions will arise, the scientific treatment of which will be of great importance for the implementation of future HATs. Thus, further research in this area is needed to address the challenges and unanswered questions associated with HMTS. Solving them will open doors to applying hybrid systems in diverse setups, thus leveraging the advantages of both, human and agent members, as human-AI multi-team systems.

## Author contributions

VH had the idea for this perspective article and took the lead in writing it. MR, AS, and FK contributed to the discussions and writing of this paper. All authors contributed to the writing and review of the manuscript and approved the version submitted.
