# Supplementary material for: Human-AI teams—Challenges for a team-centered AI at work
Source: Front Artif Intell. 2023 Sep 27;6:1252897. doi: 10.3389/frai.2023.1252897 (PMC10565103; doi:10.3389/frai.2023.1252897)
Supplement: Supplementary file 1 [file Table_1.PDF]

Supplementary Material

Human-AI Teams – Challenges for a Team-Centered AI at Work

Vera Hagemann*, Michèle Rieth, Amrita Suresh, Frank Kirchner

*** Correspondence:**
Vera Hagemann: vhagemann@uni-bremen.de

Requirements of Human-AI Teaming and their Definitions		
**Requirements**	**Definitions**	**Example References**
Responsiveness of the agents	the agents are able to align their goals and interaction strategies to the shifting goals and intentions of others as well as the environment	Lyons et al., 2022
Sound and comprehensive SA between humans and agents	SA means collecting information from systems, tools, humans, agents, and environments, interpreting this information and anticipating future states	McNeese et al., 2021
Closed-loop communication	verifying accurate message understanding through feedback: statement, repetition, reconfirmation	Salas et al., 2005
Natural communication in verbal language	speech communication in the language of the team members	Bogg et al., 2021
Interpositional knowledge	understanding of the tasks and needs of all team members to develop an understanding of the impact	Smith-Jentsch et al., 2001

	of one's actions on the actions of other team members and vice versa	
Comprehensive and up-to-date mental model of the agents	cognitive representations of system states, tasks, and processes, and help humans and agents to describe, explain, and predict situations	Mathieu et al., 2000
Explicit and implicit coordination	explicit coordination refers to communication directly focused on managing dependencies and synchronizing actions whereas implicit coordination means dependency management without dedicated or purposeful communication regarding synchronization	Schneider et al., 2021
Agents need agency	have control over their actions and the decision authority to execute these actions; helps humans to see technology as a teammate rather than as a tool to be used	Lyons et al., 2022
Flexible decision-making authority	authority dynamically shifting among the humans and agents in response to complex and changing situations	Calhoun, 2022; Schraagen et al., 2022
Resilience of the system	agents can adapt to changing processes and tasks	Lyons et al., 2022
Mutual performance monitoring	humans and agents keep track of each other while performing their own tasks	Paoletti et al., 2021

	to detect and prevent possible mistakes at an early stage	
Backup behavior in the team	the discretionary help from other human or artificial team members	Salas et al., 2005
Collective orientation of all members	the tendency to which team members value the input from others and prioritize the collective goals and interests of the entire team, rather than solely focusing on individual objectives	Hagemann et al., 2021; Salas et al., 2005
Mutual trust	shared belief that team members will perform their roles and protect the interests of their teammates	Salas et al., 2005
Cognitive competence	allows them to grasp the intentions of their teammates	Demiris, 2007, Trick et al., 2019
Predictive power	how well the model can predict the outcome of its decisions based on the situation, experience and team behavior	Raileanu et al., 2018
Reinforcement learning system design	class of machine learning algorithms, wherein the agent receives either a reward or a penalty depending on the favorability of the outcome of a particular action	Taylor, 2021
Semantic communication	content and meaning will be exchangeable between humans and agents rather than bits and bytes	Kirchner, 2020

Organized hierarchical team structure	a clear arrangement of roles and positions within a team, where humans and agents are assigned specific responsibilities and authority levels to enhance coordination and effectiveness	Vezhnevets et al., 2017
Team orientation	intelligently adapt to the situation and team requirements, in a collabo-rative, rather than a dominant or submissive manner; task involvement, information sharing, strategizing, and participatory goal setting.	Salas et al., 2005
Communication diversity	requiring agents to engage in various forms of communication, such as language, gestures, and emotions	Arriaga et al., 2017; Wang et al., 2019, Xia et al., 2019

Supplementary Table 1. Overview of the mentioned requirements for human-AI teaming, their definitions and example references.
